# Validity of Galaxy Watch for Estimating Energy Expenditure During Intermittent Running: Cross-Sectional Study

**DOI:** 10.2196/83090

**Published:** 2026-03-18

**Authors:** Alexandre Reis Pires Ferreira, Allan Inoue, Ramon Ludman Martins Barbosa, Cassio Hayek, Mateus Reis, João Arthur Alcântara, Augusto Hirao Shigueoka, Marcelo Rodrigues dos Santos

**Affiliations:** 1 Sidia Institute of Science and Technology, Av. Autaz Mirim, 2211 - Distrito Industrial I, Manaus, 69075-155, Brazil, 55 92 3212-3444

**Keywords:** energy expenditure, calories, exercise, smartwatch, wearables

## Abstract

**Background:**

Smartwatches have gained popularity for their potential to provide accurate measurements of various physiological parameters. However, the validity of energy expenditure (EE) across different smartwatch models remains a topic of ongoing investigation. Discrepancies between results obtained from different models and gold standard methods are particularly critical across varying exercise intensities and types, as validation studies have demonstrated overestimation when wearable activity monitors are compared with indirect calorimetry.

**Objective:**

This study investigated the accuracy of 2 versions of the Samsung smartwatch (Galaxy Watch [GW] 6 and 7) in measuring EE during intermittent moderate-intensity running exercises, using indirect calorimetry as the gold standard method.

**Methods:**

This study included 148 healthy adults, comprising 80 men and 68 women. Participants performed intermittent treadmill running, consisting of walking at 5 km·h⁻¹ for 1 minute and running between 8 and 16 km·h⁻¹ for 2 minutes, based on participant preference, for a total duration of 27 minutes. The GW6 and GW7 models were used and EE was measured by indirect calorimetry using a wearable portable metabolic gas analysis system (K5; Cosmed), which is considered a gold standard method.

**Results:**

No statistically significant differences were found between the GW models and the K5. The K5 showed a mean EE of 213.60 (SD 43.04) kilocalories, compared with 219.53 (SD 35.70) kilocalories for the GW6 and 202.67 (SD 47.42) kilocalories for the GW7 (all *P*>.05). Good Spearman correlations (0.63‐0.70) and moderate intraclass correlation coefficients (0.65‐0.74) were found. Mean absolute percentage error values ranged from 10.10% to 12.55%. Bland-Altman analysis revealed limits of agreement for all comparisons (K5 vs GW6 and GW7, −61.93 to 65.80 kcal).

**Conclusions:**

The GW6 and GW7 devices showed moderate validity for estimating EE during intermittent running exercises, demonstrating the suitability of the GW as a low-cost and practical wearable option for daily physical activities.

## Introduction

The accurate measurement of energy expenditure (EE) during physical activity is essential for both research and practical applications in sports science, fitness, and health monitoring. Wearable technology, such as smartwatches, has become increasingly popular due to its convenience, ease of use, and ability to provide real-time data. Among these devices, smartwatches have gained popularity for their potential to offer accurate measurements of various physiological parameters, including heart rate, step count, and EE [1,2]. However, the validity and reliability of EE across different smartwatch models remain a topic of ongoing investigation.

Recent advances in sensor technology and algorithm development have enabled smartwatches to estimate EE by combining data from accelerometers, heart rate monitors, and other sensors. Despite these advancements, questions persist regarding the accuracy of these devices compared with established gold standard methods, such as indirect calorimetry and doubly labeled water techniques [1,3,4]. This discrepancy is particularly critical across varying exercise intensities and types, where the accuracy of EE estimation may fluctuate. Validation studies have demonstrated that wearable activity monitors tend to overestimate EE when compared with indirect calorimetry [5-7], highlighting the need for accurate EE estimation to ensure effective fitness monitoring and possible research applicability.

Interdevice reliability differences in EE estimation were observed between Fitbit (Google LLC) smartwatch models depending on the type of exercise performed, with intraclass correlation coefficients (ICCs) ranging from 0.48 for jogging to 0.97 for walking. These values were compared with indirect calorimetry, which had ICCs ranging from 0.58 to 0.89 [6]. Le et al [1] evaluated the validity of the Apple Watch Series 6, Garmin FENIX 6, and Huawei Watch GT 2e for estimating EE during outdoor walking and running among young, lean Chinese adults. For walking and running activities, compared with indirect calorimetry, the Huawei Watch GT 2e demonstrated lower mean absolute percentage error (MAPE) values for EE estimation (9.9% and 11.9%, respectively), whereas Apple (19.8% and 24.4%, respectively) and Garmin (32% and 21.8%, respectively) showed higher MAPE values. For ICCs, higher values were observed for the Apple Watch during walking and running (0.821 and 0.74, respectively), followed by Huawei (0.760 and 0.698, respectively) and Garmin (0.216 and 0.594, respectively).

The Polar Grit X Pro smartwatch was considered valid for EE estimation across military training exercises (ICC, 0.8) when compared with indirect calorimetry assessment [3]. Similarly, Roos et al [8] analyzed the validity of 3 different smartwatches compared with indirect calorimetry at 2 different running intensities. The results showed that the accuracy of the smartwatches was intensity-dependent when compared at 30%, 50%, 70%, 90%, and 110% of the velocity associated with peak oxygen consumption (VO_2 peak_), with MAPE values ranging from 10.9% to 49.3% and the greatest variation observed at 110% of VO_2 peak_ between smartwatch estimates and criterion measurements.

Limitations in smartwatch validation research include the absence of regulatory standards [4], small sample sizes [1,3], heterogeneity of methods [3-5], and “black box” variables, such as algorithms and sensor updates. As demonstrated in previous studies, the accuracy of EE estimation and the heterogeneity of results across devices underscore the need for comprehensive validation methodologies, appropriate statistical approaches [3,5,8], and larger sample sizes [1,3].

The validity and accuracy of EE metrics provided by smartwatches are critical, as they are frequently used as decision-making data for physical activity and control of caloric intake [4,5]. In addition, the literature lacks three critical elements of smartwatch EE validity: (1) validity of EE measures in intermittent running activities, (2) accuracy across different models of the same smartwatch, and (3) more representative and larger sample sizes. Moreover, the validity of smartwatches is company- and model-dependent, as different companies tend to use distinct algorithms by patent issues, and the software may be upgraded in subsequent versions. In this context, it is important to conduct validity studies among growing companies in the health technology segment, such as Samsung, which has released at least 7 generations of smartwatches aimed at the health market.

This study aimed to evaluate the accuracy of 2 models of Samsung Galaxy Watch (GW; GW6 and GW7) in measuring EE during exercise, using gold standard methods as a reference (indirect calorimetry), during intermittent moderate-intensity running exercises. By assessing the performance of these devices, this study aimed to provide insights into their utility for both scientific research and everyday use among individuals seeking to monitor their health and fitness levels.

## Methods

### Study Design

This was a cross-sectional, within‐subject study designed to compare estimated EE from 2 models of the Samsung GW (the GW6 and the GW7) with criterion measures of EE obtained using a wearable metabolic gas analyzer (K5; Cosmed) during intermittent running. Reporting followed the Guidelines for Reporting Reliability and Agreement Studies [9]. Three K5 gas analyzers were used to perform the measurements. Before any treadmill activity began, all participants were screened by a physician and a treadmill stress test was performed with a 12-lead electrocardiogram. After screening on a separate day, participants attended the laboratory to perform the tests. Skin color was measured using a Mexameter MX18 spectrophotometer (Courage+Khazaka electronic GmbH) and categorized between 1 and 6 following the Fitzpatrick Classification scale [10]. The exercise protocol consisted of intermittent walking and running.

### Participants

This study recruited 148 healthy adults, comprising 80 men and 68 women. The participants were physically active and engaged in at least 150 minutes of moderate-intensity activity and/or 75 minutes of vigorous activity per week. Inclusion criteria were (1) being physically active, defined as engaging in at least 150 minutes of light- to moderate-intensity exercise, and (2) being aged 18 to 59 years. Exclusion criteria were (1) musculoskeletal injuries of the lower limbs, (2) cardiovascular or lung diseases, and (3) use of medications that impact heart rate response, such as beta blockers.

### Ethical Considerations

All participants were informed about the study procedures and provided written informed consent. This study was conducted in accordance with the ethical principles contained in the Declaration of Helsinki and complied with national regulations governing research involving human participants (466/12 of the National Health Council). The experimental procedures followed established ethical standards and were approved by the Investiga - Institutos de Pesquisa ethics committee (CAE 62031722.4.0000.5599). The privacy and confidentiality of all research participants and their data and identities were maintained throughout the study. All personal identifiers were removed from the dataset, and data were anonymized to ensure compliance with ethical standards. Access to the data was restricted to the research team, and all data were stored securely in accordance with institutional guidelines. In accordance with the legal regulations of our country, participants in this study did not receive monetary compensation. However, they were provided with reimbursement for transportation and meal expenses as a token of appreciation for their time and contributions.

### Exercise Protocol

To test the validity of the GW6 and the GW7 under standardized laboratory conditions, participants performed an activity of intermittent walking and running for a total duration of 27 minutes, consisting of walking at 5 km·h⁻¹ and running between 8 and 16 km·h⁻¹, based on the participant’s preference, on a commercial treadmill (Star Trac, E-TRxe). The protocol consisted of a 2-minute walking warm-up, followed by 7 repetitions of 2 minutes of running and 1 minute of walking, and concluded with an additional 4 minutes of walking recovery (27 min total).

### Criterion Measure

The K5 portable metabolic cart was used as the criterion method for estimating EE. This device is considered a valid and reliable system for measuring metabolic variables [1,11]. Device set up, calibration, and data extraction were conducted via OMNIA Metabolic software (version 1.6.10) (Cosmed). For the calculation of EE, oxygen uptake (VO_2_) was measured continuously, breath by breath, throughout the entire 27-minute protocol. Two pieces of K5 equipment were used for the data collection. Before each session, all K5 equipment was calibrated according to the manufacturer’s instructions.

### Wearable Activity Monitors

Two GW models (GW6 and GW7) were used and connected to a smartphone (A22; Samsung Electronics), and all EE data was exported using the Samsung Health app. In cases in which activity data required trimming, full health data were exported via the Samsung Health app, and the raw CSV file was imported into Microsoft Excel (version 2310) for further analysis and cleaning. Participants’ weight, height, date of birth, and wrist orientation were entered into each watch before the activity protocol. The watches were positioned on the left arm, just above the wrist (2 cm above the ulnar styloid process), as per manufacturer instructions. The GWs were set to record the activity protocol as a workout, with watches being started and stopped at the beginning and at the end of the activity. The GWs were set to the “treadmill” mode.

### Statistical Analysis

The sample size was estimated a posteriori using a custom Python script, with an α level of .05 and statistical power of 0.90, identifying a minimum sample size of 29 participants for the GW6, 18 participants for the GW7, and 21 participants for both GW models. The power calculated using the actual sample size was 1.0 for all comparisons. Additionally, the sample size was considered adequate based on the recommendations of Chevance et al [12], who consider sample sizes of more than 50 participants to be reasonably large. Moreover, the sample size in this study was larger than that of previous comparable studies of wrist-worn health-tracking devices, in which participant numbers ranged from 11 to 60 [1,3,8,13,14]. Data distribution was inspected, and outliers were identified using boxplot analysis and removed if they exceeded the standard error limits. The lower limit was defined as the first quartile minus 1.5 × the interquartile range (IQR), and the upper limit was defined as the third quartile plus 1.5 × the IQR. Data normality was verified by the Shapiro-Wilk test.

Two‐sided paired *t* tests were used to compare the K5 with the GW6, the K5 with the GW7, and the K5 with the GW6 and GW7. Significant differences (*P*<.05) were interpreted using Cohen *d* effect sizes, with the threshold defined as trivial (<0.20), small (0.20‐0.59), moderate (0.60‐1.19), large (1.20‐1.99), and very large (2.00‐4.00) [15]. To evaluate systematic differences, correlation, precision, and agreement between the wearables and the criterion measure, MAPE (calculated as [actual – predicted]/actual × 100), ICC (2-way random-effects model, absolute agreement, single measurement), Spearman correlation, and Bland-Altman analysis were performed.

The MAPE value was interpreted as poor (≥20%), moderate (10.1%-19.9%), good (3.1%-10.0%), and excellent (0%-3%) [1,14,16]. Spearman correlation coefficients were interpreted as negligible (0.00-0.09), weak (0.10-0.39), moderate (0.40-0.69), strong (0.7-0.89), and very strong (0.90-1.00) [17]. Finally, ICC values were interpreted as poor (<0.50), moderate (0.50-0.74), good (0.75-0.89), and excellent (≥0.90) [18].

Statistical analyses were performed using JASP software (version 0.18.3; JASP Team) and a custom Microsoft Excel (version 2506) spreadsheet [19]. Statistical significance was set at *P*≤.05 (2-tailed). Based on the current literature, results were considered valid when values were at least moderate for ICCs, Spearman correlation, and MAPE.

## Results

Participants had a mean age of 29.98 (SD 9.01) years, a mean height of 1.65 (SD 0.09) meters, a mean body mass of 65.53 (SD 11.18) kilograms, and a mean skin pigmentation score of 3.60 (SD 1.11) according to the Fitzpatrick classification scale. Energy expenditure during intermittent running is shown in Table 1.

**Table 1.** Energy expenditure during intermittent running with the Galaxy Watch (GW), measured with the Cosmed K5 metabolic gas analyzer.

Devices (N=148)	Energy expenditure (kcal), mean (SD)	95% CI
K5_GW6 (n=79)	218.63 (39.70)	227.52-209.74
K5_GW7 (n=69)	207.84 (46.20)	218.94-196.74
K5_All (n=148)	213.60^a^ (43.04)	220.59-206.61
GW6 (n=79)	219.53 (35.70)	227.53-211.54
GW7 (n=69)	202.67^a^ (47.42)	214.06-191.28
GW_All (n=148)	211.67 (42.28)	218.54-204.80

a Statistical significance for the Shapiro-Wilk test (*P*≤.05).

Table 2 shows systematic differences, correlations, and measure of precision. For systematic differences, a paired *t* test was conducted to compare EE values between the K5 and the GW6. No statistically significant differences were found between the K5 versus the GW6, the K5 and GW7, or the K5 and the combined GW6 and GW7 (GW6+GW7). Spearman correlation indicated moderate associations between the K5 and GW6 and between the K5 and the combined GW6 and GW7, and a strong association between the K5 and GW7. Moreover, moderate ICC and MAPE values were found for all comparisons.

**Table 2.** Spearman correlation (SC), intraclass correlation (ICC), and mean absolute percentage error (MAPE) of energy expenditure during intermittent running with the Galaxy Watch (GW) and Cosmed K5 metabolic gas analyzer.

Devices (criterion, practical)	SC (95% CI)	ICC (95% CI)	MAPE, mean (SD)
K5, GW6	0.63^a^ (0.47-0.75)	0.65 (0.55-0.74)	10.10 (9.34)
K5, GW7	0.70^a^ (0.55-0.80)	0.74 (0.66-0.81)	12.55 (8.78)
K5_GW6+GW7	0.67^a^ (0.57-0.75)	0.71 (0.62-0.78)	10.86 (9.06)

a Statistical significance for the Spearman correlation at *P*≤.05.

**Figure 1.**
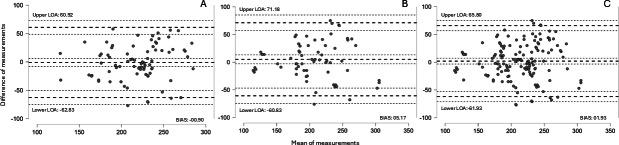
Bland-Altman LOA between the (A) Cosmed K5 metabolic gas analyzer and GW6, (B) Cosmed K5 metabolic gas analyzer and GW7, and (C) Cosmed K5 metabolic gas analyzer and GW6 and GW7. GW: Galaxy Watch; LOA: limits of agreement.

Figure 1 shows Bland-Altman plots with bias and limits of agreement between criterion and practical measurements, presented as mean, upper, and lower calorie values.

## Discussion

### Principal Findings

The Samsung GW6 and GW7 models were evaluated for validity in estimating EE during intermittent, moderate-intensity exercise. The results showed a moderate to strong correlation and no statistically significant difference in paired *t* tests when compared with the gold standard of indirect calorimetry. Additionally, both the GW6 and GW7 models achieved a MAPE value of less than 15%, which indicates a moderate absolute error.

Existing literature reveals variability in smartwatch validation results due to differing algorithms, hardware generations, and study designs. For instance, LaMunion et al [20] found significant differences between 5 consumer devices, including 4 smartwatches, when compared with the Cosmed K4b^2^ portable metabolic gas analysis system during semistructured daily activities. In contrast, the GW6 and GW7 models in our study showed moderate validity under intermittent running conditions compared with the K5 gas analyzer, likely due to advancements in technology and the structured exercise protocol used in this study.

Previous studies have reported EE estimation with mean ICC values ranging from −0.30 to 0.97 [3,5,6,21], 95% CIs ranging from −0.89 to 0.98 [5,6,21,22], and MAPE values ranging from 1.3% to 114.7% [21-23]. Among the studies, Wahl et al [21] analyzed 11 different wearable watches in walking and running protocols at speeds of 4.3, 7.2, 10.1, and 13.0 km·h^-1^. Mean ICC values greater than 0.70 were observed for only 4 smartwatches, whereas ICC values greater than 0.50 were observed for 6 smartwatches. Mean absolute percentage error values ranged from 1.3% to 56.0%. For ICCs, 17 of 66 measurements were greater than 0.5, and only 3 of these were obtained under intermittent conditions (mean ICC range 0.54-0.74). For MAPE, 24 of 66 measurements were less than 10%, and only 5 of these were obtained under intermittent conditions, ranging from −9.2% to 5.6%.

The moderate ICC and MAPE values for the GW6 and the GW7 compared with the K5 were considered acceptable indicators of validity, as previously reported [1,5,12,13]. Associations between variables were also assessed using Spearman correlation, demonstrating moderate values for the GW6 group and the combined GW6 and GW7 group, and strong values for the GW7 group. Moreover, the database comprised both men and women, resulting in a heterogeneous sample. As expected, heterogeneous samples may influence accuracy metrics, as smartwatch EE estimates have been shown to differ across different demographic groups [24].

It is important to highlight that different types of exercises can affect the variability of smartwatch accuracy [1,21]. The assessment of EE in this study differs from previous investigations primarily in terms of device performance and testing conditions. Earlier research has demonstrated generally low validity for consumer wearable activity monitors in estimating EE (particularly under both constant and intermittent running protocols) [21]. Wahl et al [21] highlighted consistent overestimation of EE at lower speeds and underestimation at higher speeds. While not perfectly valid, the Garmin, Fitbit, and Withings (Withings SA) wearable watches generally showed better validity with small to moderate MAPE values (1.3%‐21.2%), particularly at faster velocities (10.1 km·h^−1^ and 13.0 km·h^−1^) and in outdoor conditions. Devices such as the Garmin Vivofit, Garmin Vivosmart, Garmin Vivoactive, Fitbit Charge, and Fitbit Charge HR exhibited moderate to good ICCs, suggesting a relatively better correlation with the criterion measure compared with other brands. However, even for these devices, the limits of agreement were still insufficiently narrow, indicating considerable variability and imprecision.

For the intermittent protocol, which simulates conditions in sports such as soccer, the Fitbit Charge, Garmin Vivoactive, Garmin Vivosmart, and Polar Loop showed a relatively small MAPE (<5.6%). However, most other devices, including the Withings Pulse Ox (wrist or hip), Garmin Forerunner 920XT, Garmin Vivofit, Beurer AS80, and Bodymedia Sensewear, primarily underestimated EE during this dynamic activity. This reinforces the concern that most commercially available wearable activity monitors lack the accuracy required to reliably estimate EE in real-world exercise scenarios, especially among athletes.

The magnitude of the limits of agreement for these wearable activity monitors indicates that, although average error during intermittent running can be small to moderate in relative terms, individual-level EE estimates can still deviate by several hundred kilocalories in a typical session, which is likely too large for precise real-world monitoring, especially in athletes [21]. During intermittent exercise, rapid changes in speed, direction, and intensity violate the steady state assumptions many EE algorithms rely on, leading to larger MAPE values and wider limits of agreement than during constant-velocity running [21,25].

When compared with criterion measures, several studies have demonstrated that wearable activity monitors underestimate EE in both laboratory and outdoor tests [2,8,13,23,26], demonstrating a mean value of approximately 3 kilocalories and percentage errors ranging from −21.27% to 14.76% [23]. Importantly, few studies have analyzed the EE validity of Samsung smartwatches, with the Samsung Gear models being the most tested smartwatches [26]. Likewise, Sun et al [13] identified EE overestimation in the Apple Watch Series 6 when compared with a breath-by-breath gas analyzer (MetaMax 3B; CORTEX Biophysik GmbH) across different running velocities (6.4-11.2 km·h⁻¹), despite high MAPE values (–6.61 to 53.24).

One limitation of this study is that only 1 brand of smartwatch was tested. While this could be seen as a constraint, it also presents an advantage by allowing for a direct comparison of technological improvements for a specific model within the same brand. By examining the evolution of technology in this specific area, we gain valuable insights into how advancements have positively impacted the accuracy of these devices over time. Another limitation is that the study tested EE measurement during 1 type of exercise and only in healthy, lean people. This restricts the scope of our findings, as the accuracy of smartwatch EE estimates can vary across different forms of physical activity and physical characteristics. Future research should address this limitation by evaluating the performance of these devices across a wider range of populations and exercise modalities, such as resistance training, high-intensity interval training, and low-intensity steady-state exercise. This would provide a more comprehensive understanding of the accuracy of devices and limitations in various exercise contexts. Another limitation of this study is that 2 different K5 devices were used in data collection. Although manufacturer calibration was performed immediately after the data collection sessions, no between-device validation was conducted. Future research should use a single device throughout the data collection period or perform between-device validation.

### Practical Implications

The following points summarize the practical implications of the present findings.

- Both the GW6 and the GW7 can be used for EE estimation in intermittent running exercises, providing valid data for athletes and fitness enthusiasts.
- Advancements in the GW7 highlight the importance of algorithm and hardware updates and underscore the need for continuous development in the wearable activity monitor industry.
- This study helps consumers make more informed decisions regarding which smartwatch model is best suited for accurate fitness tracking.
- The results of this study set a benchmark for future validation studies, promoting a more standardized approach in wearable technology research.

### Conclusions

Both the GW6 and GW7 models showed moderate validity for estimating EE during intermittent running exercises. These findings support the growing reliability of newer smartwatches for EE estimation in structured exercise settings. Given their relatively low cost and practicality, GWs emerge as a suitable wearable option for daily physical activity where EE is of interest.

## Abbreviations

EE: energy expenditure
GW: Galaxy Watch
ICC: intraclass correlation coefficient
MAPE: mean absolute percentage error
